# Detection of tick-borne bacteria and babesia with zoonotic potential in *Argas* (*Carios*) *vespertilionis* (Latreille, 1802) ticks from British bats

**DOI:** 10.1038/s41598-018-20138-1

**Published:** 2018-01-30

**Authors:** Jizhou Lv, Maria del Mar Fernández de Marco, Hooman Goharriz, L. Paul Phipps, Lorraine M. McElhinney, Luis M. Hernández-Triana, Shaoqiang Wu, Xiangmei Lin, Anthony R. Fooks, Nicholas Johnson

**Affiliations:** 1 Chinese Academy of Inspection and Quarantine, Beijing, 100176 P.R. China; 2 Animal and Plant Health Agency, Woodham Lane, Surrey, KT15 3NB UK; 3 Institute of Infection and Global Health, University of Liverpool, Liverpool, L69 7BE UK; 4 Faculty of Health and Medical Sciences, University of Surrey, Guildford, Surrey GU2 7XH UK

## Abstract

Ticks host a wide range of zoonotic pathogens and are a significant source of diseases that affect humans and livestock. However, little is known about the pathogens associated with bat ticks. We have collected ectoparasites from bat carcasses over a seven year period. Nucleic acids (DNA and RNA) were extracted from 296 ticks removed from bats and the species designation was confirmed in all ticks as *Argas* (*Carios*) *vespertilionis*. A subset of these samples (n = 120) were tested for the presence of zoonotic pathogens by molecular methods. *Babesia* species, *Rickettsia* spp., within the spotted fever group (SFG), and *Ehrlichia* spp. were detected in ticks removed from 26 bats submitted from 14 counties across England. The prevalence of *Rickettsia* spp. was found to be highest in *Pipistrellus pipistrellus* from southern England. This study suggests that the tick species that host *B. venatorum* may include the genus *Argas* in addition to the genus *Ixodes*. As *A. vespertilionis* has been reported to feed on humans, detection of *B. venatorum* and SFG *Rickettsia* spp. could present a risk of disease transmission in England. No evidence for the presence of flaviviruses or Issyk-Kul virus (nairovirus) was found in these tick samples.

## Introduction

Bats (Chiroptera) are widely distributed across the United Kingdom (UK), with 17 species that breed indigenously^1^. Bats are recognized as reservoirs or carriers of numerous species of viruses, bacteria and protozoan parasites, many with zoonotic potential to infect humans^2,3^. They also host a range of ectoparasites that could play a role in the transmission of pathogenic organisms. During recent decades, increasing urbanisation and the adaptation of bats to urban habitats has increased the opportunities for contact between bats and bat-associated ticks with humans and domestic animals^3–5^. Ticks are obligate hematophagous arthropods that are considered second only to mosquitoes as vectors of pathogens that cause disease in humans and animals^6^. Bats are subject to parasitism by a number of specialized tick species^7^. Bats form roosts that they return to on a daily basis and can become infested with ticks. The adaptation of ticks to such environments is referred to as nidicolous or nest-dwelling behaviour. Nidicolous bat ticks include *Argas vespertilionis* (also known as *Carios vespertilionis*), *Ixodes vespertilionis* and *I. ariadnae*. Other tick species associated with bats include *I. simplex*, *I. ricinus* and *Dermacentor reticulatu*s^3,8^. Both *I. ricinus* and *A. vespertilionis* are widely distributed across the UK^9^. These ticks can also bite humans and have the potential to transmit pathogens between bats and human^4,10^. The epidemiological significance for the transmission of diseases to humans from bats and bat ticks has become increasingly recognized although limited by our understanding of bat tick distribution and pathogen associations. A range of vector-borne pathogens have been detected in bat ticks including piroplasms (*Babesia vesperuginis*, *B. crassa*, *B. canis*, *Theileria capreoli*, and *T. orientalis*), *Borrelia* (*Borrelia burgdorferi*, *Bo. CPB1*), *Rickettsia* spp., *Ehrlichia* spp. and Issyk-kul virus^3,4,11^. *Bo. burgdo*rferi sensu lato, the causative agent of Lyme disease, was detected in *A. vespertilionis* collected between 1896 and 1994 from England and Wales^11^. *B. vesperuginis* is hypothesized to be a vector-borne *Babesia* transmitted by *A. vespertilionis* that is virulent to some bat species^12^.

To estimate the burden of tick-borne diseases and host exposure to bat tick bites in the UK, detailed information about distribution and abundance of bat ticks and bat tick-borne diseases is critical. There have been no comprehensive reports regarding this field in the UK bat tick population comparable to similar studies in continental Europe^3^. In order to assess the risk of pathogen transmission from bat-associated ticks, the distribution and species abundance of ticks collected from bats across the UK has been investigated and tested for a range of tick-borne pathogens.

## Results

### Tick infestations of bats

**Figure 1:**
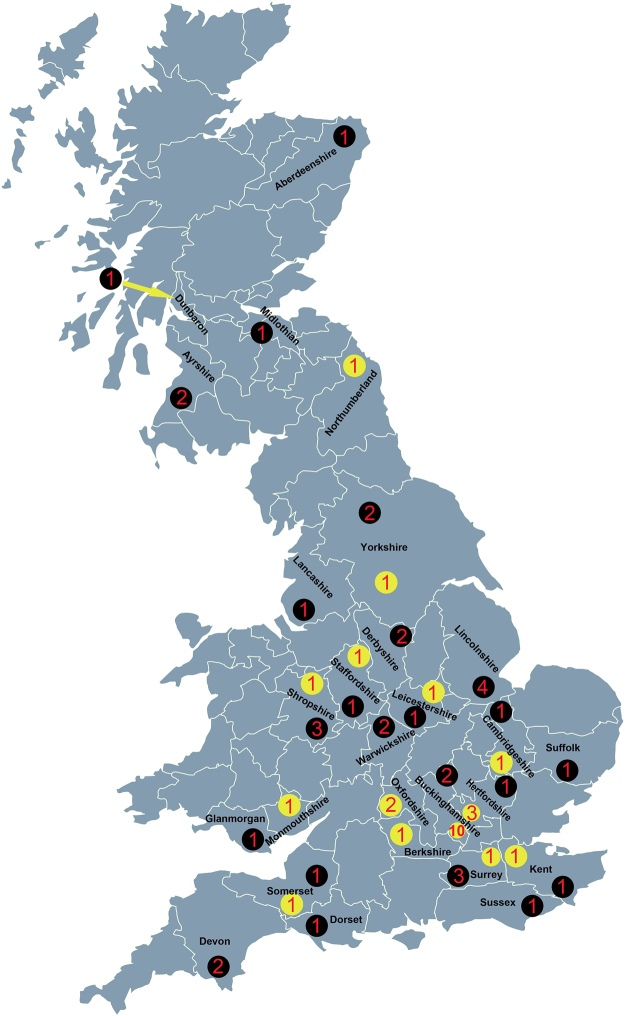
Map of Great Britain showing locations where bats were submitted and the ectoparasites sampled. The collection sites for bat ticks are marked with yellow dots, for other parasites such as fleas and mites with black dots. Numbers in yellow or black dots indicate the number of bats sampled from each county. This figure is not included in the Creative Commons licence for the article; all rights reserved. Taken from the Beijing Zcool Internet Technology Co., Ltd.

**Figure 2:**
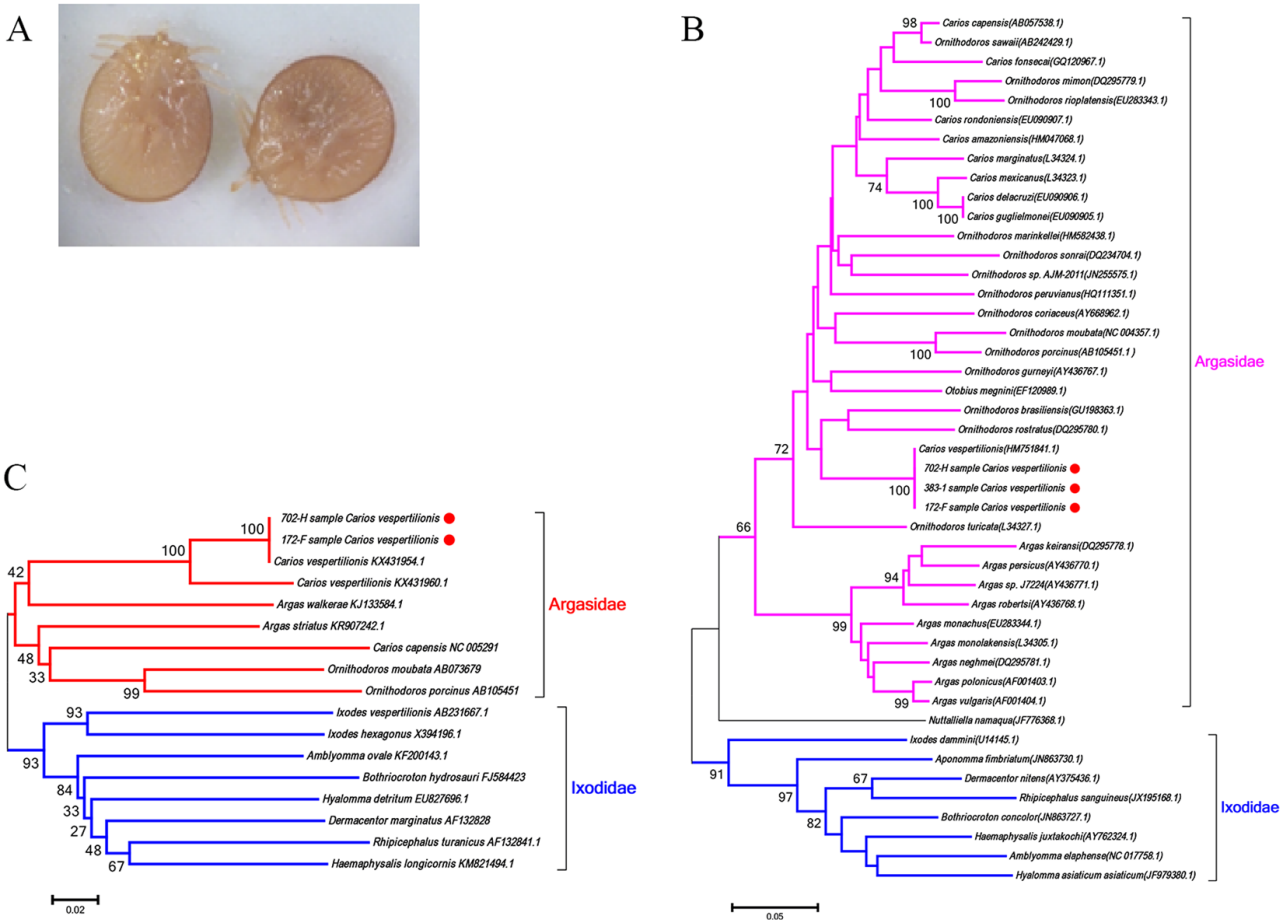
Species identification of bat ticks through morphology and Neighbor Joining phylogenetic analyses based on COI and 16S rRNA. (**A**) A representative image of bat ticks removed from a UK bat. (**B**) Neighbor Joining phylogenetic analysis based on partial tick 16S rRNA sequence, (**C**) Neighbor Joining phylogenetic analysis based on partial tick COI sequence. Bootstrap values are indicated at the nodes. Scale bar indicates the degree of divergence represented by a given length of branch. The red dots indicate the sequences acquired in this study.

In order to assess the pathogens associated with UK bats, ectoparasites were collected from 62 infested bats submitted to APHA between 2007 and 2013 (total submissions were 7,606). The bat species were identified as *Myotis daubentonii* (n = 8), *Pipistrellus pipistrellus* (n = 38), *Plecotus auritus* (n = 5), *Myotis natteri* (n = 2), *Eptesicus serotinus* (n = 1), *Myotis mystacinus* (n = 3), *Nyctalus noctula* (n = 2) and *Rhinolophus hipposideros* (n = 2) (Supplementary Table S1). The species of one bat could not be determined due to carcass decomposition. The locations from where bats were submitted are shown in Fig. 1. A total of 296 ticks were removed from 26 bats and identified as larvae of *A. vespertilionis* based on morphology (Fig. 2A). Phylogenetic analyses using sequences derived from the cytochrome oxidase subunit I (COI) and 16 S ribosomal RNA (rRNA) confirmed this designation (Fig. 2B,C). No other tick species were identified in this study. Hundreds of bats were submitted from Scotland, and five were infested with ectoparasites but not ticks (Supplementary Table S1, Fig. 1). Of the nine species of bats included in the study, *P. pipistrellus*, *Pl. auritus*, and *M. daubentonii* were hosts of *A. vespertilionis* (Table 1).

**Table 1:** Summary of ticks collected from UK bats between 2007 and 2013.

Tick	Bat species (number of ticks per number of bats)							
Species	*Pipistrellus pipistrellus*	*Plecotus auritus*	*Myotis daubentonii*	*Myotis nattereri*	*Eptesicus serotinus*	*Myotis mystacinus*	*Nyctalus noctula*	*Rhinolophus hippsideros*
*Argas vespertilions*	267/36	23/5	6/8	0/2	0/1	0/3	0/2	0/2

### Detection of piroplasms in bat ticks

**Figure 3:**
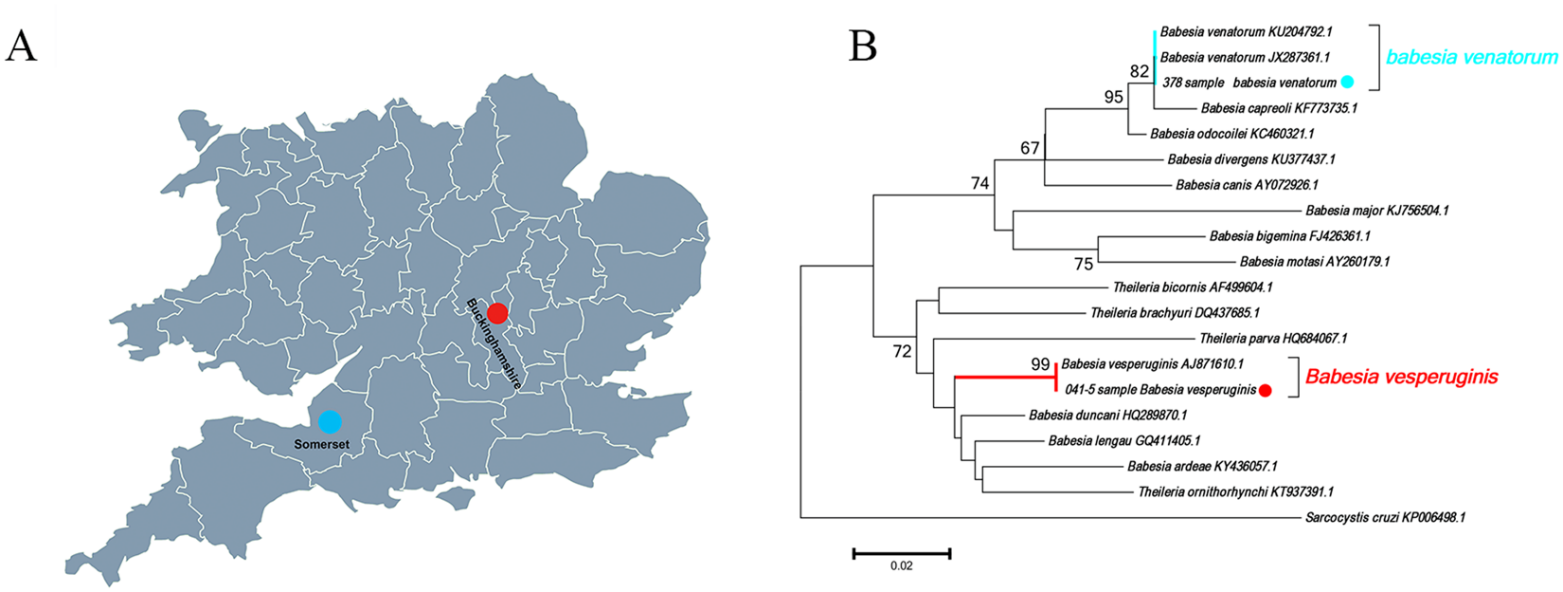
Detection and analysis of piroplasm DNA detected in ticks removed from UK bats. (**A**) Map showing the sampling sites of piroplasm-positive bat ticks. The red dot indicates the site of *B. vesperuginis* positive *A. vespertilionis* and the blue dot indicates the site of *B. venatorum* positive *A. vesperitilionis*. This figure is not included in the Creative Commons licence for the article; all rights reserved. Taken from Beijing Zcool Internet Technology Co., Ltd. (**B**) Neighbor Joining phylogenetic analysis based on partial 18S rRNA sequence of *Babesia* spp. Bootstrap values are indicated at the nodes. Scale bar indicates the degree of divergence represented by a given length of branch. The red dot indicates the sequence of *B. vesperuginis* and the blue dot indicates the sequence of *B. venatorum* acquired in this study.

DNA sequences of piroplasms were detected in three bat ticks collected from Buckinghamshire and Somerset (Table 2, Fig. 3A). DNA sequence analysis of the amplicons derived from two *A. vespertilionis* removed from bat 041/2008 submitted from Buckinghamshire showed 100% identity with *B. vesperuginis* (AJ871610). A DNA sequence obtained from a bat tick removed from a *Pl. auritus* bat from Somerset (378/2013) shared 99% identity with *B. venatorum* (KU204792) and 98% with *B. capreoli* (KF773735). Phylogenetic analysis based on a partial sequence of 18 S rRNA (Fig. 3B) confirmed that the *Babesia* sequence derived from bat 041/2008 clustered with *B. vesperuginis* sequences while the *Babesia* sequence obtained from bat 378/2013 clustered with a clade including both *B. venatorum* (KU204792, JX287361) and *B. capreoli* (KF773735).

**Table 2:** Molecular analyses of bat ticks for the presence of piroplasms.

Tick species	Piroplasm positive/all analysed ticks	Results of sequencing (length, % identity, sample number)	Bat hosts	Locations of piroplasm positive ticks
*Argas vespertilionis*	3/120	*Babesia vesperuginis* (426 bp, 99%, 2)	*Pipistrellus pipistrellus*	Buckinghamshire
		*Babesia venatorum* (426 bp, 99%, 1)	*Plecotus auritus*	Somerset

### Detection of *Rickettsia* spp

**Figure 4:**
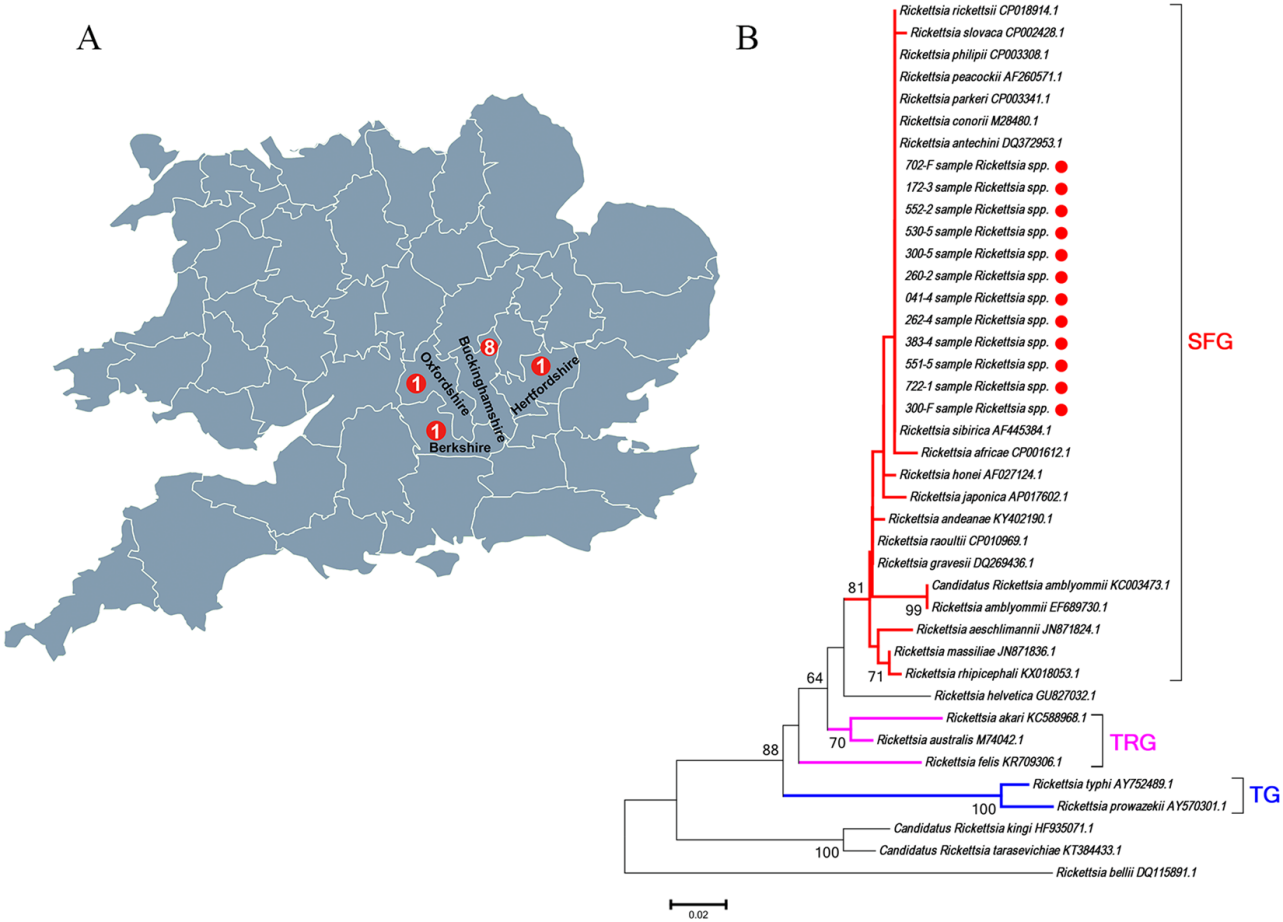
Detection and analysis of *Rickettsia* spp. from UK bat ticks. (**A**) Map showing the sampling sites of *Rickettsia* spp. positive bat ticks. The red dots indicate the sites of *Rickettsia* spp positive *A. vespertilionis*. This figure is not included in the Creative Commons licence for the article; all rights reserved. Taken from Beijing Zcool Internet Technology Co., Ltd. (**B**) Neighbor Joining phylogenetic analysis based on a partial sequence of the 17 K Da protein gene of *Rickettsia* spp. Bootstrap values are indicated at the nodes. Scale bar indicates the degree of divergence represented by a given length of branch. The red dot indicates the sequence of *Rickettsia* spp. acquired in this study. Numbers in red dots indicate the number of bats with *Rickettsia* spp. positive ticks, sampled from each county.

In previous reports, SFG *Rickettsiae* spp. such as *R. helvetica* and *R. raoultii* were detected in *I. ricinus*, *Haemaphysalis punctata* and *D. reticulatus* ticks in England and Wales^13,14^. In this study, *Rickettsia* spp. were detected in 16 bat ticks collected from 11 bats submitted from 4 counties of England (Buckinghamshire, Oxfordshire, Hertfordshire and Berkshire) (Table 3, Fig. 4A). Fifteen *A. vespertilionis* were removed from 10 *P. pipistrellus*, while one was removed from one *Pl. auritus* (Table 3). DNA sequence analysis of the 16 amplicons demonstrated that they shared 100% identity with the 17 kDa protein gene of *Rickettsia* spp. such as *Rickettsia sibirica* (MF002549.1) and *Rickettsia conorii* (MF002513.1). A phylogenetic tree based on a partial sequence of the 17 kDa gene of representative *Rickettsia* is shown in Fig. 4B. This demonstrates that all the *Rickettsia* sequences detected in this study clustered with species in the spotted fever group (SFG) including *R. conorii* (M28480), *R. rickettsii* (CP018914), and *R. africae* (CP001612).

**Table 3:** Molecular analyses of bat ticks for the presence of *Rickettsia*.

Tick species	*Rickettsia* positive/all analysed ticks	Results of sequencing (length, % identity, sample number)	Bat hosts, host number	Locations of positive ticks
*Argas vespertilionis*	16/120	Rickettsia spp. (434 bp, 99%, 1)	*Plecotus auritus*, 1	Buckinghamshire
		Rickettsia spp. (434 bp, 99%, 11)	*Pipistrellus pipistrellus*, 7	Buckinghamshire
		Rickettsia spp. (434 bp, 99%, 1)	*Pipistrellus pipistrellus*, 1	Oxfordshire
		Rickettsia spp. (434 bp, 99%, 2)	*Pipistrellus pipistrellus*, 1	Hertfordshire
		*Rickettsia spp*. (434 bp, 99%, 1)	*Pipistrellus pipistrellus*, 1	Berkshire

### Detection of *Ehrlichia/Anaplasma* spp

**Figure 5:**
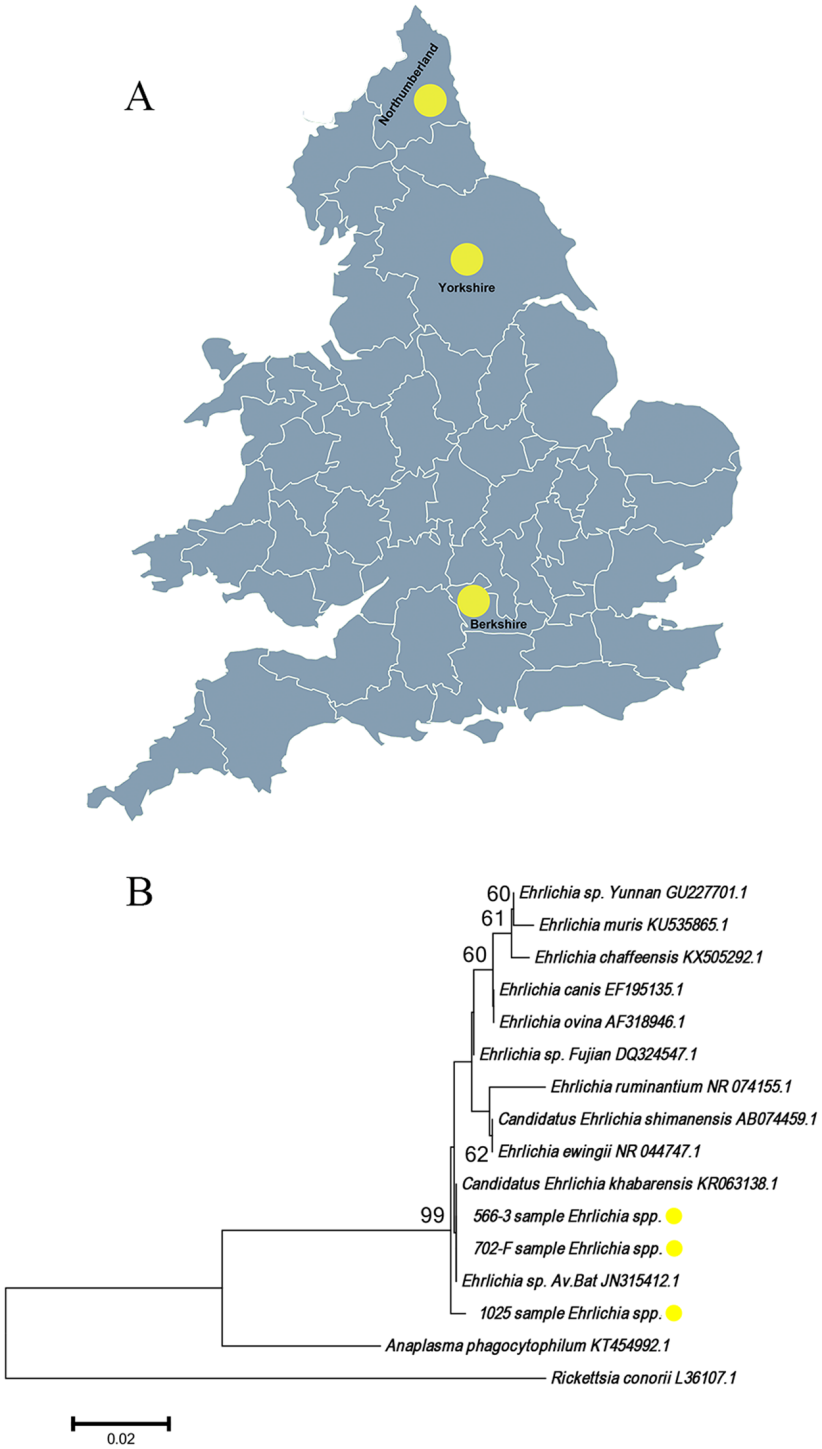
Detection and analysis of *Ehrlichia*/*Anaplasma* spp. from UK bat ticks. (**A**) Map showing the sampling sites of *Ehrlichia* spp. positive *A. vespertilionis* (yellow dots). This figure is not included in the Creative Commons licence for the article; all rights reserved. Taken from Beijing Zcool Internet Technology Co., Ltd. (**B**) Neighbor Joining phylogenetic analysis based on a partial 16S rRNA sequence of *Ehrlichia* spp. Bootstrap values are indicated at the nodes. Scale bar indicates the degree of divergence represented by a given length of branch. The yellow dot indicates the sequence of *Ehrlichia* spp. acquired in this study.

*Ehrlichia canis* has been detected in a dog with no history of travel outside the UK in a previous study^15^. In this study, DNA sequences of *Ehrlichia*/*Anaplasma* spp. were detected in 5 bat ticks collected from 3 bats (*P. pipistrellus*) submitted from 3 counties of England (Yorkshire, Northumberland and Berkshire) (Table 4, Fig. 5A). DNA sequence analysis of the 5 amplicons showed that they shared 99% sequence identity with the 16S rRNA of *E. yunnan* (GU227701.1). A phylogenetic tree based on 16S rRNA of the representative *Ehrlichia*/*Anaplasma* spp. is shown in Fig. 5B. This shows that all the *Ehrlichia* sequences detected in this study clustered in a clade that includes *E. canis* (EF195135.1) and uncultured *Ehrlichia* spp. (JN315412.1).

**Table 4:** Molecular analyses of bat ticks for the presence of *Ehrlichia*.

Tick species	*Ehrlichia* positive/all analysed ticks	Results of sequencing (length, % identity, sample number)	Bat hosts, host number	Locations of positive ticks
*Argas vespertilionis*	5/120	*Ehrlichia spp*. (675 bp, 99%, 1)	*Pipistrellus pipistrellus*, 1	Berkshire
		*Ehrlichia spp*. (675 bp, 99%, 1)	*Pipistrellus pipistrellus*, 1	Northumberland
		*Ehrlichia spp*. (675 bp, 99%, 3)	*Pipistrellus pipistrellus*, 1	Yorkshire

### Detection of *Borrelia* spp., *Coxiella burnetii*, Issyk-kul virus and Flaviviruses

A total of 120 *A. vespertilionis* were tested for the presence of *Borrelia* spp. and *Coxiella burnetii* DNA. Results were negative in all samples for these bacteria. The RNA samples prepared from 120 *A. vespertilionis* were screened for the presence of Issyk-kul virus and flaviviruses. All samples were also negative for these viruses.

## Discussion

A range of hard ticks including *I. vespertilionis*, *I. ariadnae*, *I. simplex*, *I. ricinus* and *D. reticulatus* have been reported to infest bats^3,16,17^. *I. vespertilionis* and *A. vespertilionis* have previously been reported to be associated with bats in the UK and *A. vespertilionis* is believed to be widely distributed across England and the west of Scotland^11^. In this study, *A. vespertilionis* ticks were collected from 26 bat carcasses submitted from 14 counties of England (Fig. 1). *P. pipistrellus*, in addition to being the most abundant bat species in the UK and most commonly submitted for lyssavirus testing, was also the most commonly infested with bat ticks. This bat has been reported to host *A. vespertilionis* in other European studies^18^. The infestation rate for *P. pipistrellus*, *Pl. auritus* and *M. daubentonii* are separately 7.4, 11.5 and 2 per bat and generally the average for all three bat species is 7 ticks per bat. All bat ticks collected in this study were larvae and no adults or nymphs were found. In addition to ticks, we also collected other parasites such as fleas and mites (Supplementary Table S1), but these were not investigated further.

Due to the considerable lack of data in the literature concerning the zoonotic pathogens associated with bat ticks in the UK, nucleic acid extracts of 120 specimens were tested for the presence of a range of pathogens. The role of *A. vespertilionis* as a vector or reservoir of bacterial or protozoal pathogens in France had been reported^4^. *B. vesperuginis* DNA was identified in *A. vespertilionis* collected from *P. pipistrellus* in this study. This soft tick species has been incriminated as a vector of *B. vesperuginis* in Central Europe^19^ which has been reported to infect *P. pipistrellus*, several *Myotis* spp. (including *M. daubentonii*) and *Pl. auritus* in England^12^. Of particular interest was the detection of *B. venatorum* from one larva of *A. vespertilionis* infesting *Pl. auritus* from the county of Somerset (Fig. 3). *B. venatorum* is zoonotic with *I. ricinus* and *I. simplex* as vectors of this parasite. It is noteworthy that *I. ricinus* and *I. simplex* occur on bats and this suggests that bats may be carriers of this *Babesia*^20^. This suggests that *A. vespertilionis* is a potential host of *B. venatorum* in addition to the genus *Ixodes*, and that *Pl. auritus* may be infected with *B. venatorum*. As *A. vespertilionis* is the most common bat tick worldwide and is known to bite humans^21,22^, it could potentially transmit *B. venatorum* to humans as this is a known zoonotic pathogen^23^. Alternatively, these *Argasid* ticks are positive for *Babesia* through contact with infected bats and are not biological vectors.

Species in the genus *Rickettsia* are separated into three groups: The first, an ancestral group containing *R. felis*; A second, the typhus group (TG) which includes the agent of louse-borne epidemic typhus, *R. prowazekii*, and the agent of flea-borne murine typhus, *R. typhi*; Finally a third, the SFG, whose members are associated mainly with ticks^24,25^. Based on phylogenetic analysis of a partial sequence of the 17 kDa protein, we have demonstrated that the *Rickettsia* spp. detected in *A. vespertilionis* from the UK can be classified within the SFG *Rickettsia*. The result is consistent with the findings of Socolovschi and co-workers, who showed that *Rickettsia* spp. within the SFG were detected from 3 of 5 *A. vespertilionis* collected from a human dwelling in France^4^. In our study, all of the *A. vespertilionis* ticks in which *Rickettsia* spp. were detected were obtained from bats submitted from four adjacent counties, all located in southern England. Tick-borne *Rickettsia* spp. within the SFG are associated with several human diseases in Europe including *Rickettsia conorii conorii*, the agent of Mediterranean spotted fever (MSF), *R. conorii israelensis* (Israeli spotted fever); *R. slovaca* and *R. raoultii* agents of tick-borne lymphadenopathy (TIBONEL)^26,27^. SFG *Rickettsia* such as *R. helvetica* and *R. raoultii* have been detected in *I. ricinus* and *D. reticulatus* ticks in the UK^13^. However, no human cases of infection with *Rickettsia* spp. have been reported.

The presence of *Ehrlichiae* spp. DNA was detected in *A. vespertilionis* collected from three bats in this study. *Ehrlichia*/*Anaplasma* spp. DNA has been detected from *A. vespertilionis* in France^4^ and the sequence similarity between *Ehrlichia* sp. Av bat of France and the *Ehrlichia* spp. detected in this study is over 99.5%. The species and pathogenicity of *Ehrlichia* spp. detected in the UK requires further investigation.

A single larva of *A. vespertilionis* was found attached to a dead bat infected with a *Borrelia* spp. in the UK and *A. vespertilionis* may be the source of infection^28^. Also, *Borrelia* sp.CPB1 was detected from the *A. vespertilionis* in France^4^. In 1966, *Coxiella burnetii*, the agent of Q fever, was detected in *A. vespertilionis* collected from southern Kazakhstan^29^. *Coxiella burnetii* has been reported in livestock populations of England and Wales^30^. These findings suggest that *A. vespertilionis* ticks from UK may be the vectors or reservoirs for *Borrelia* spp. and *Coxiella burnetii*. However, neither of these pathogens was detected from the 120 *A. vespertilionis* collected from bats in this study suggesting that *A. vespertilionis* ticks in UK may not be a common host for *Borrelia* spp. and *Coxiella burne*tii until further evidence is found.

Evidence for the role of *A. vespertilionis* as vectors or reservoirs of viral pathogens is limited. In 1973, Issyk-Kul virus, assigned to the *Bunyaviridae* family, was isolated from bats (*Nyctalus noctula*, *Myotis blythi* and *Vespertilio serotinus*) and *A. vespertilionis* in Kyrgyzstan^31^. Recently, a novel *Bunyavirus* was isolated from *A. vespertilionis* in Japan, sharing between 76 and 79% identity with Issyk-Kul virus^32^. A number of viruses belonging to genus *Flavivirus* has been isolated from bats and this suggests that bat ticks have the potential to be vectors for flaviviruses^33,34^. In this study we screened 120 bat ticks of UK origin for both Issyk-Kul virus and flaviviruses. We were unable to demonstrate the presence of these viruses from this cohort. Potential reasons for this include true absence of virus from this tick population although the sample size was relatively small. Alternatively, more sensitive means of detection might be required or the samples experienced degradation of nucleic acid during storage prior to testing.

In conclusion, we have detected a range of potential pathogens in ticks associated with British bats. Previous reports have indicated that *A. vespertilionis* can bite humans. This suggests that they are a potential source of pathogens for those that have close contact with bats, particularly the common pipistrelle, could be at risk of exposure and re-emphasizes the need for bat handlers to wear appropriate personal protective equipment such as gloves to avoid exposure to both bat-borne and tick-borne pathogens.

## Materials and Methods

### Ectoparasite collection and identification

Bat carcasses were submitted to the Animal and Plant Health Agency as part of passive surveillance for lyssaviruses from 2007 to 2013, in particular for European Bat Lyssavirus type 2 (EBLV-2), which has previously been detected in the UK^35^. Bat speciation was based on morphology and the locations and dates of submission recorded. Ectoparasites (ticks, mites and fleas) were removed from each carcass from a total number of 7606 bats submitted to APHA between 2007 and 2013. These were immediately stored in 75% ethanol at room temperature. Morphological identification of ectoparasites was achieved through examination using a stereo microscope (SDZ-PL, Kyowa Instruments, Japan) with reference to standard morphological keys^36^.

### Nucleic acid extraction

One hundred and twenty ticks were dried, and then washed three times with distilled water. All ticks were bisected with disposable scalpels; one half utilized for DNA extraction and the other half utilized for RNA extraction. DNA was extracted with the DNeasy Blood & Tissue kit (QIAGEN, Hilden, Germany) according to the manufacturer’s instructions. DNA was eluted in 80 μL elution buffer AE (provided with kit) and stored at −80 °C until tested. RNA was extracted with the RNeasy mini kit (QIAGEN, Hilden, Germany) according to the manufacturer’s instructions. RNA was eluted in 60 μl of RNase-free water (provided with kit) and stored at −80 °C until tested.

### Molecular identification of tick species

Sequences of the 16S rRNA and COI genes were amplified by PCR using the primers listed in supplementary Table S2. PCR amplification was carried out using GoTaq G2 Flexi DNA polymerase (Promega, WI, USA). The reaction master mix was prepared according to the manufacture’s protocol and the PCR conditions described in previous studies were used^37,38^. All PCRs were run with positive and negative controls. PCR products were separated using gel electrophoresis in 1% agarose gels, stained with SYBR Safe DNA Gel Stain (Invitrogen, CA, USA) and visualized under ultra-violet light.

### Detection of DNA pathogens

All primer sequences are provided in supplementary Table S2. Piroplasms were detected using primers PIROA and PIROB^39^. *Rickettsia* spp. were detected using a nested set of primers targeting the gene sequence of the 17 kDa protein^40^. *Ehrlichia*/*Anaplasma* spp. were detected using a hemi-nested primer set targeting the 16 S rRNA gene^41^. *Borrelia* spp. were detected using a primer pair that targeted the flagellin gene^42^. *Coxiella burnetti* was detected using a primer pair targeting the Cb IS 1111 element^43^. The reaction master mix was prepared according to the manufacture’s protocol and the PCR conditions described in previous studies were used.

### Detection of RNA viruses

Reverse transcription was carried out with M-MLV reverse transcriptase **(**Promega, WI, USA) in a reaction volume of 40 μL, which included 18 μL of extracted RNA, 8 μL 5× RT buffer, 2 μL dNTP (10 mM), 3 μL DTT (0.1 M), 4 μL M-MLV reverse transcriptase (200 U/μL), 1 μL RNasin (40 U/μL), 2 μL 10× hexanucleotide mix, and 2 μL molecular grade water.

Detection of Flavivirus RNA was attempted using the hemi-nested -PCR targeting the RNA-dependent RNA polymerase gene, as described previously^44^. In brief, 5 μL cDNA was utilized as template for each reaction. The reaction master mix was prepared according to the manufacture’s protocol and the PCR conditions described in previous studies were used.

Detection of the *Nairovirus*, Issyk-Kul virus, was attempted using a hemi-nested PCR targeting the S segment of the virus genome using primers described in supplementary Table S2. After denaturation at 95 °C for 2 minutes (min), the reactions were cycled 45 times at 95 °C for 30 seconds (s), 50 °C for 30 s, and 72 °C for 50 s, followed by an elongation step at 72 °C for 7 min, finally the reactions were cooled down to 4 °C. The hemi-nested PCR reaction utilized 1 μl of the first PCR product as template with primers BUNV-F2/BUNV-R2 (Table S2). After denaturation at 95 °C for 2 min, the reactions were cycled 45 times at 95 °C for 30 s, 60 °C for 30 s, and 72 °C for 40 s, followed by an elongation step at 72 °C for 7 min, finally the reactions were cooled to 4 °C.

### Sequence Analysis

DNA amplicons of the correct size were purified and sequenced as previously described^39^. Representative sequences were submitted to GenBank (supplementary Table S3). DNA sequences were assembled using Lasergene version 12.1 (DNASTAR) and edited in MEGA 5.0^45^. Sequence alignments were conducted using ClustalW within MEGA 5.0, using default parameters (open gap penalty = 10.0, extend gap penalty = 5.0) before subsequently being checked by visual inspection. Genetic distances were calculated based on the K2P model for all pair-wise comparisons in the matrix using MEGA^46^. Bootstrapping (1000 replicates) was utilized to estimate node support. Pairwise deletion was used for gaps/missing data. Based on K2P distances, phylogenetic trees were constructed with the combined data sets of all major tick genera using the Neighbor-Joining method. For COI analysis, all codon positions and non-codon sites were tested combined.

### Data availability

All data discussed in the manuscript is included in the paper.

## Electronic supplementary material

Supplementary Tables

## References

[1] Helversen, O.V., Nill, D. & Dietz, C. Bats of Britain, Europe and Northwest Africa. *Bloomsbury Specialist* (2009).

[2] Calisher CH, Childs JE, Field HE, Holmes KV, Schountz T (2006). Bats: important reservoir hosts of emerging viruses. Clin Microbiol Rev..

[3] Hornok S (2016). DNA of piroplasms of ruminants and dogs in *Ixodid* bat ticks. PLoS One.

[4] Socolovschi, C., Kernif, T., Raoult, D. & Parola, P. *Borrelia*, *Rickettsia*, and *Ehrlichia* species in bat ticks, France, 2010. *Emerg Infect Dis*. **18**, 1966–1975 (2012). 10.3201/eid1812.111237 PMC3557878 23171714

[5] Krauel JJ, LeBuhn G (2016). Patterns of bat distribution and foraging activity in a highly urbanized temperate environment. PLoS One..

[6] de la Fuente, J. *et al*. Tick-pathogen interactions and vector competence: Identification of molecular drivers for tick-borne diseases. *Front Cell Infect Microbiol*. **7**, 114 (2017). 10.3389/fcimb.2017.00114 PMC5383669 28439499

[7] Dantas-Torres F (2012). Description of a new species of bat-associated argasid tick (Acari: Argasidae) from Brazil. J Parasitol..

[8] Durden LA, Beckman KB, Gerlach RF (2016). New records of ticks (Acari: Ixodidae) from dogs, cats, humans and some wild vertebrates in Alaska: invasion potential. J Med Entomol..

[9] Jameson LJ, Medlock JM (2011). Tick surveillance in Great Britain. Vector Borne Zoonotic Dis..

[10] Jaenson TGT (1994). Geographical distribution, host association, and vector roles of ticks (Acari: Ixodidae, Argasidae) in Sweden. J. Med. Entomol..

[11] Hubbard MJ, Baker AS, Cann KJ (1989). Distribution of *Borrelia burgdorferi* s.l. spirochaete DNA in British ticks (*Argasidae* and *Ixodidae*) since the 19^th^ century, assessed by PCR. Med Vet Entomol..

[12] Gardner RA, Molyneux DH (1987). Babesia vesperuginis: natural and experimental infections in British bats (Microchiroptera). Parasitology.

[13] Tijsse_Klasen E (2011). First detection of spotted fever group *Rickettsia*e in *Ixodes ricinus* and *Dermacentor reticulatus* ticks in the UK. Epidemiol Infect..

[14] Tijsse-Klasen E (2013). Spotted fever group rickettsiae in Dermacentor reticulatus and Haemaphysalis punctata ticks in the UK. Parasit Vectors..

[15] Wilson HE, Mugford AR, Humm KR, Kellett-Gregory LM (2013). *Ehrlichia canis* infection in a dog with no history of travel outside the United Kingdom. J Small Anim Pract..

[16] Hornok S (2015). Contributions to the morphology and phylogeny of the newly discovered bat tick species, *Ixodes ariadnae* in comparison with *I. vespertilionis* and *I. simplex*. Parasit Vectors..

[17] Burazerovic J (2015). Ticks (Acari: Argasidae, Ixodidae) parasitizing bats in the central Balkans. Exp Appl Acarol..

[18] Hornok S (2015). High degree of mitochondrial gene heterogeneity in the bat tick species *Ixodes verspertilionis*, *I. ariadnae* and *I. simplex* from Eurasia. Parasit Vectors..

[19] Hornok S (2017). Molecular investigations of the bat tick *Argas vespertilionis* (Ixodida: Argasidae) and *Babesia vesperuginis* (Apicomplexa: Piroplasmida) reflect “bat connection” between Central Europe and Central Asia. Exp Appl Acarol..

[20] Venclikova K, Mendel J, Betasova L, Hubalek Z, Rudolf I (2015). First evidence of *Babesia venatorum* and *Babesia capreoli* in questing *Ixodes ricinus* ticks in the Czech Republic. Ann Agric Environ Med..

[21] Wilhelmsson P (2013). *Ixodes ricinus* ticks removed from humans in Northern Europe: seasonal patterns of infestation, attachment sites and duration of feeding. Parasit Vectors..

[22] Estrada-Pena A, Jongejan F (1999). Ticks feeding on humans: a review of records on human-biting Ixodoidea with special reference to pathogen transmission. Exp Appl Acarol..

[23] Jiang JF (2015). Epidemiological, clinical, and laboratory characteristics of 48 cases of “*Babesia venatorum*” infection in China: a descriptive study. Lancet Infect Dis..

[24] Brouqui P, Parola P, Fournier PE, Raoult D (2007). Spotted fever rickettsioses in southern and eastern Europe. FEMS Immunol Med Microbiol..

[25] Punda-Polic V (2002). Detection and identification of spotted fever group rickettsiae in ticks collected in southern Croatia. Exp Appl Acarol..

[26] Parola P, Paddock CD, Raoult D (2005). Tick-borne rickettsioses around the world: emerging diseases challenging old concepts. Clin Microbiol Rev..

[27] Parola P (2009). Rickettsia slovaca and R. raoltii in tick-borne Rickettsioses. Emerg Infect Dis..

[28] Evans NJ, Bown K, Timofte D, Simpson VR, Birtles RJ (2009). Fatal borreliosis in bat caused by relapsing fever spirochete, United Kingdom. Emerg Infect Dis..

[29] Zhmaeva ZM, Pchelkina AA, Belashova VS (1966). [Spontaneous infection of *Argas vespertilionis* with *Rickettsia burnetii* in the south of Kazakhstan]. Med Parazitol..

[30] Jones RM (2010). Detection of *Coxiella burnetii* in placenta and abortion samples from British ruminants using real-time PCR. Vet Rec..

[31] Lvov DK (1973). “Issyk-Kul” virus, a new arbovirus isolated from bats and *Argas* (*Carios*) *vespertilionis* (Latr, 1802) in the Kirghiz S.S.R. Brief report. Arch Gesamte Virusforsch..

[32] Oba *et al*. A novel *Bunyavirus* from the soft tick, *Argas vespertilionis* in Japan. *J Vet Med Sci*. **78**, 443–445. 10.1292/jvms.15-0536 PMC4829514 26498534

[33] de Lamballerie X (2002). Genome sequence analysis of Tamana bat virus and its relationship with the genus Flavivirus. J Gen Virol..

[34] Tajima S, Takasaki T, Matsuno S, Nakayama M, Kurane I (2005). Genetic characterization of Yokose virus, a flavivirus isolated from the bat in Japan. Virology..

[35] Wise, E. L. *et al*. Passive surveillance of United Kingdom bats for lyssaviruses (2005–2015). *Epidemiol Infect*. **145**, 2445–2457 (2017). 10.1017/S0950268817001455 PMC9148805 28737119

[36] Hillyard, P. S. *Arga*s (*Cario*s) *vespertilioni*s (Latreille, 1802) in Ticks of North-West Europe. 142–3 (Field Studies Council, 1996).

[37] Krakowetz CN, Dergousoff SJ, Chilton NB (2010). Genetic variation in the mitochondrial 16S rRNA gene of the American dog tick, *Dermacentor variabilis* (Acari: Ixodidae). J Vector Ecol..

[38] Folmer O, Black M, Hoeh W, Lutz R, Vrijenhoek R (1994). DNA primers for amplification of mitochondrial cytochrome c oxidase subunit I from diverse metazoan invertebrates. Mol Mar Biol Biotechnol..

[39] Fernández de Marco M (2017). Emergence of *Babesia canis* in southern England. Parasit Vectors.

[40] Anstead CA, Chilton NB (2013). A novel *Rickettsia* species detected in Vole Ticks (*Ixodes angustus*) from Western Canada. Appl Environ Microbiol..

[41] Teshale S (2016). Molecular detection of *Anaplasma* species in questing ticks (Ixodids) in Ethiopia. Asian Pac J Trop Dis..

[42] Park HS (2004). Evaluation of groEL gene analysis for identification of Borrelia burgdorferi sensu lato. J Clin Microbiol..

[43] Capuano F, Proroga YTR, Mancusi A, Perugini AG, Berri M (2016). Evaluation of DNA preparation methods combined with different PCR-based assays for *Coxiella burnetii* detection in milk. Large Anim Rev..

[44] Abdel-Shafy S, Allam NAT (2013). Quantitative real-time RT-PCR detection of flaviviruses associated with camel ticks in Egypt. Global Veterinaria..

[45] Tamura K (2011). MEGA5: molecular evolutionary genetics analysis using maximum likelihood, evolutionary distance, and maximum parsimony methods. Mol Biol Evol..

[46] Aliabadian M, Kaboli M, Nijman V, Vences M (2009). Molecular identification of birds: performance of distance-based DNA barcoding in three genes to delimit parapatric species. PLoS One..

