# Supplementary material for: Detection of tick-borne bacteria and babesia with zoonotic potential in Argas (Carios) vespertilionis (Latreille, 1802) ticks from British bats
Source: Sci Rep. 2018 Jan 30;8:1865. doi: 10.1038/s41598-018-20138-1 (PMC5789838; doi:10.1038/s41598-018-20138-1)
Supplement: Supplementary file 1 — Supplementary Tables [file 41598_2018_20138_MOESM1_ESM.pdf]

Detection of tick-borne bacteria and babesia with zoonotic potential in *Argas (Carios) vespertilionis* (Latreille, 1802) ticks from British bats

Jizhou Lv, Maria del Mar Fernández de Marco, Hooman Goharriz, L. Paul Phipps, Lorraine M. McElhinney, Luis M. Hernández-Triana, Shaoqiang Wu, Xiangmei Lin, Anthony R. Fooks, Nicholas Johnson

Supplimentary Table S1: Ectoparasites specimens collected from bats submitted from across the UK between 2007 and 2013.

Sample number (year)	Hosts	County	*Argas vespertilionis* (ticks)		No. of collected unidentified Non-ticks parasites
			No. of collected specimens	***No. of screened specimens***	
96 (2007)	*Myotis daubentonii*	Buckinghamshire	6 larva	4 larva	
103 (2007)	*Pipistrellus pipistrellus*	Suffolk			3 specimens
134 (2007)	*Pipistrellus pipistrellus*	Staffordshire			2 specimens
163 (2007)	*Pipistrellus pipistrellus*	Buckinghamshire	14 larva	6 larva	
167 (2007)	*Myotis mystacinus*	Derbyshire			7 specimens
171 (2007)	*Pipistrellus pipistrellus*	Surrey	5 larva	3 larva	
172 (2007)	*Plecotus auritus*	Buckinghamshire	22 larva	5 larva	
306 (2007)	*Nyctalus noctula*	Derbyshire			9 specimens
384 (2007)	*Pipistrellus pipistrellus*	Warwickshire			19 specimens
388 (2007)	*Pipistrellus pipistrellus*	Yorkshire			10 specimens
408 (2007)	*Pipistrellus pipistrellus*	Hertfordshire			3 specimens
418 (2007)	*Myotis daubentonii*	Lincolnshire			11 specimens
584 (2007)	*Pipistrellus pipistrellus*	Yorkshire			8 specimens
685 (2007)	*Pipistrellus pipistrellus*	Staffordshire	11 larva	6 larva	
862 (2007)	*Pipistrellus pipistrellus*	Aberdeenshire			13 specimens
873 (2007)	*Pipistrellus pipistrellus*	Shropshire	15 larva	6 larva	
952 (2007)	*Pipistrellus pipistrellus*	Midlothian			21 specimens

041 (2008)	*Pipistrellus pipistrellus*	Buckinghamshire	21 larva	6 larva	
224 (2008)	*Pipistrellus pipstrellus*	Monmouthshire	7 larva	6 larva	
260 (2008)	*Pipistrellus pipistrellus*	Oxfordshire	29 larva	6 larva	
262 (2008)	*Pipistrellus pipistrellus*	Hertfordshire	12 larva	6 larva	
300 (2008)	*Pipistrellus pipistrellus*	Buckinghamshire	14 larva	8 larva	
312 (2008)	*Pipistrellus pipistrellus*	Cambridgeshire			16 specimens
383-A (2008)	*Pipistrellus pipistrellus*	Buckinghamshire	22 larva	6 larva	
383-B (2008)	*Pipistrellus pipistrellus*	Buckinghamshire			4 specimens
551 (2008)	*Pipistrellus pipistrellus*	Buckinghamshire	15 larva	6 larva	
552 (2008)	*Pipistrellus pipistrellus*	Buckinghamshire	6 larva	4 larva	
575 (2008)	*Myotis nattereri*	Shropshire			11 specimens
585 (2008)	*Pipistrellus pipistrellus*	Oxfordshire	12 larva	6 larva	
702 (2008)	*Pipistrellus pipistrellus*	Berkshire	30 larva	4 larva	
722 (2008)	*Pipistrellus pipistrellus*	Buckinghamshire	17 larva	6 larva	
922 (2008)	*Myotis daubentonii*	Ayrshire			9 specimens
1127 (2008)	*Pipistrellus pipistrellus*	Cambridgeshire	12 larva	6 larva	
070 (2009)	*Pipistrellus pipistrellus*	Lincolnshire			3 specimens
147 (2009)	*Myotis daubentonii*	Ayrshire			10 specimens
290 (2009)	*Eptesicus serotinus*	Somerset			2 specimens
292 (2009)	*Pipistrellus pipistrellus*	Kent			7 specimens
570 (2009)	*Myotis nattereri*	Dunbarton			3 specimens
800 (2009)	*Pipistrellus pipistrellus*	Kent	5 larva	4 larva	

932 (2009)	*Myotis daubentonii*	Dorset			4 specimens
1046 (2009)	*Plecotus auritus*	Buckinghamshire			1 specimens
1091 (2009)	*Pipistrellus pipistrellus*	Sussex			1 specimens
013 (2010)	*Plecotus auritus*	Leicestershire			2 specimens
089 (2010)	*N/A*	Lincolnshire			1 specimens
326 (2010)	*Pipistrellus pipistrellus*	Lancashire			13 specimens
417 (2010)	*Pipistrellus pipistrellus*	Hertfordshire	1 larva	1 larva	
428 (2010)	*Myotis mystacinus*	Glamorgan			3 specimens
530 (2010)	*Pipistrellus pipistrellus*	Buckinghamshire	7 larva	5 larva	
979 (2010)	*Myotis daubentonii*	Surrey			5 specimens
978 (2010)	*Myotis daubentonii*	Surrey			2 specimens
2019 (2010)	*Myotis daubentonii*	Warwickshire			3 specimens
1071 (2010)	*Pipistrellus pipistrellus*	Leicestershire	1 larva	1 larva	
628 (2011)	*Myotis mystacinus*	Shropshire			2 specimens
866 (2011)	*Pipistrellus pipistrellus*	Lincolnshire			2 specimens
886 (2011)	*Plecotus auritus*	Surrey			8 specimens
1059 (2011)	*Nyctalus noctula*	Shropshire			1 specimens
1025 (2011)	*Pipistrellus pipistrellus*	Northumberland	1 larva	1 larva	
04 (2012)	*Rhinolophus hipposideros*	Devon			8 specimens
115 (2012)	*Rhinolophus hipposideros*	Devon			13 specimens
566 (2012)	*Pipistrellus pipistrellus*	Yorkshire	8 larva	6 larva	
45 (2013)	*Pipistrellus pipistrellus*	Hertfordshire	2 larva	1 larva	

378 (2013)	*Plecotus auritus*	Somerset	1 larva	1 larva	
Total number			296 larva	120 larva	142 specimens

Supplementary Table S2: Primers used to detect the pathogens in bat ticks collected in UK.

Target	Gene Target	Primer pairs	Amplicon(bp)
Arthropod species	CO1	HCO1490:<br>5’-GTTCAACAAATCATAAAGATATTGG-3’<br>LCO2198:<br>5’-TAAACTTCAGGGTGACCAAAAAATCA-3’	675
Arthropod species	16SrRNA	16S-1:<br>5’-CCACAGCAATTTAAAAAATCATTGAGCAG-3’<br>16S+1:<br>5’-CCGGTCTGAACTCAGATCAAGT-3’	454
Piroplasm spp.	18S rRNA	PIROA:<br>5’-AATACCCAATCCTGACACAGGG-3’<br>PIROB:<br>5’-TTAAATACGAATGCCCCCAAC-3’	426
Rickettsia spp.	17 kDa	17K-5: 5’-GCTTTACAAAATTCTAAAAACCATATA-3’<br>17K-3:5’ –TGTCTATCAATTCACAACTTGCC-3’<br>17kD1: 5’-GCTCTTGCAACTTCTAT GTT-3’<br>17kD2: 5’-CATTGTTCGTCAGGTTGGCG-3’<br>First round:17K-5/17K-3<br>Second round: 17kD1/17kD2	434
Ehrlichia / Anaplasma spp.	16S rRNA	EHR 16SD:5’ -GGTACCYACAGAAGAAGTCC-3’<br>EBR3: 5’-TTGTAGTCGCCATTGTAGCAC-3’<br>EBR2: 5’-TGCTGACTTGACATCATCCC-3’<br>First round: EHR 16SD/ EBR3<br>Second round: EHR 16SD/ EBR2	925
Borrelia spp.	Flagellin	F1a:5’-GCAGTTCAATCAGGTAACGG-3’<br>F2a:5’-AGGTTTTCAATAGCATACTC-3’	565
Issyk-kul virus	S SEGMENT	BUNV-F2:5’-TGTGGGCAAGAGGmcttagatcctc-3’<br>BUNV-R1: 5’-AAGCATGCCAGCATAYTGAAGATG-3’<br>BUNV-R2: 5’-AACACTAGTTAAMAGCTACAGATG-3’<br>First round: BUNV-F2/BUNV-R1<br>Second round: BUNV-F2/BUNV-R2	320
Coxiella burnetii	Cb IS 1111 element	CF:5’-tatgtatccaccgtagccagtc-3’<br>CR:5’-cccaacaacacctccttattc-3’	687
Flavivirus	RNA-dependentRNA polymerase	PF1S: 5’-TGYRTBTAYAACATGATGGG-3’<br>PF2R-bis: 5’-GTGTCCCAICCNGCNGTRTC-3’<br>PF3S: 5’-ATHTGGTWYATGTGGYTDGG-3’<br>First round: PF1S / PF2R-bis<br>Second round: PF3S / PF2R-bis	197

Supplementary Table S3: GenBank Accession numbers for sequences obtained in this study.

Species	Sample codes	Target gene	Accession numbers
Argas vespertilionis	702-H	COI	MF510173
Argas vespertilionis	172-F	COI	MF510174
Argas vespertilionis	702-H	16S rRNA	MF510175
Argas vespertilionis	172-F	16S rRNA	MF510176
Argas vespertilionis	383-1	16S rRNA	MF510177
Babesia venatorum	378	18S rRNA	MF510178
Babesia vesperuginis	041-5	18S rRNA	MF510179
Rickettsia spp.	041-4	17 kDa	MF510180
Rickettsia spp.	260-2	17 kDa	MF510181
Rickettsia spp.	262-4	17 kDa	MF510182
Rickettsia spp.	300-5	17 kDa	MF510183
Rickettsia spp.	383-4	17 kDa	MF510184
Rickettsia spp.	530-5	17 kDa	MF510185
Rickettsia spp.	551-5	17 kDa	MF510186
Rickettsia spp.	552-2	17 kDa	MF510187
Rickettsia spp.	722-1	17 kDa	MF510188
Rickettsia spp.	172-3	17 kDa	MF510189
Rickettsia spp.	300-F	17 kDa	MF510190
Rickettsia spp.	702-F	17 kDa	MF510191
Ehrlichia spp.	702-F	16S rRNA	MF510192
Ehrlichia spp.	566-3	16S rRNA	MF510193
Ehrlichia spp.	1025	16S rRNA	MF510194
